# Do Similar Objects Have Similar Grasp Positions?

**DOI:** 10.3390/s24237735

**Published:** 2024-12-03

**Authors:** Qi Sun, Lili He

**Affiliations:** School of Computer Science and Technology, Zhejiang Sci-Tech University, Hangzhou 310018, China; sunqi@zstu.edu.cn

**Keywords:** grasping, shape descriptor, deep neutral network (DNN)

## Abstract

In robotic grasping tasks, shape similarity has been widely adopted as a reference in grasp positions prediction for unknown objects. However, to the best of our knowledge, the issue “do similar objects have similar grasp positions?” has not been quantitatively analyzed before. This work aims to confirm or disprove the question by analyzing the relationship between the object shape similarity and grasp positions similarity. To this end, we constructed a similarity-estimation plane (SE-Plane), whose horizontal and vertical axes indicate the objects similarity and grasp similarity, respectively. Then, the proof of the issue is equal to the confirmation of the inference that “the points with higher objects similarity accordingly own higher grasps similarity in the proposed SE-Plane”. We adopted several classical shape descriptors and two kinds of widely recognized deep neural network (DNN) architectures as objects similarity strategies. Furthermore, we employed the widely adopted intersection-over-union (IoU) of grasp anchors to measure the grasp similarity between objects. The experiments were carried out on a dozen objects with commonly seen primitive shapes selected from two well-known open grasp datasets: Cornell and Jacquard. It was found that the IoU values of grasp anchors are generally proportional to those of objects similarity in the SE-Plane. In addition, we obtained several primitive shapes from the commonly seen shapes, which are more suitable references in grasp positions prediction for unknown objects. We also constructed a realistic object dataset that included the objects with commonly seen primitive shapes. With the IoU prediction strategy learned from Cornell and Jacquard, the IoU predicted for realistic objects yielded similar results in the proposed SE-Plane. These discussions indicate that “similar objects have similar grasp positions” is reasonably correct. The proposed SE-Plane presents a new strategy to measure the relationship between objects similarity and grasp similarity.

## 1. Introduction

Robotic grasping of unknown objects is an open problem in the robotic community. It was reported that human grasp behavior for known and familiar objects depends on object identifications and object presentations [1,2]. As for an unknown object, the appearance features provide information about the object’s parts and their respective affordances, which are then processed by the brain in a spatial stream to assist in the action selection process. Additionally, they contribute to a trade-off between appearance and identification for grasping [2]. Inspired by human’s visual pathways for perception and action, early researchers consider grasping unknown objects to be a problem due to the shape, texture, and weight that is to be handled by a subsequent fine controller. In the early stages, shape similarity was widely used for robotic grasp positions prediction of unknown objects, especially when the objects’ materials and weights were unknown [2,3,4,5,6,7,8].

In contrast to the conventional shape feature descriptors devised by humans, a deep neural network (DNN) is capable of predicting the optimal grasp position from object image presentations. This is achieved by analyzing the vast number of images with labeled anchors, which indicate the positions and orientations of potential grasps [9,10]. The deep neural network is able to achieve high performance in grasp prediction from image presentations due to the knowledge gained from the labeled grasp anchors [11,12]. Furthermore, some researchers combined the deep neural network with shape feature descriptors in the grasping tasks for two-dimensional (2D) [13] and three-dimensional (3D) [14,15,16,17] grasp position prediction of unknown objects.

However, to our best knowledge, a fundamental question underlying these studies—do similar objects have similar grasp positions—has not been discussed before.There have been some researchers who combined shape categories [18], shape features [2,19,20], and shape contours [5,15] in the grasping anchors’ prediction. However, in these works, how the objects grasping positions were influenced by their shapes was not discussed. This work aims to confirm or disprove the issue by analyzing the relationship between the objects’ shape similarity and grasp similarity. The contributions of this work include the following:

- We analyze the correlation between the grasping positions and the similarity of the objects for a dozen commonly seen shapes. This is a basic and important issue in the research of robotic grasps, which has not been discussed before.
- We construct a similarity-estimation plane (SE-Plane), whose horizontal and vertical axes indicate the objects similarity and grasp similarity, respectively. Then, the proof of the target question is converted to confirm the object pair relationships in the SE-Plane; namely, the object pair with higher objects similarity is expected to have higher grasp similarities as well.
- We adopt several classical shape descriptors and the widely recognized deep neural network (DNN) architectures as objects similarity strategies and use THE intersection-over-union (IoU) of grasp anchors to measure the grasp similarity between objects. With the correlation analysis between the grasping positions and the objects similarity for different objects in the SE-Plane, we found some primitive shapes which have higher correlations between the grasp positions and objects similarity. We believe the discussions and the discoveries in this work provide valuable information about the robotic grasping of unknown objects.

The remainder of this work is organized as follows: the related works are introduced in Section 2; the details of the calculation and analysis of the correlation between the grasp positions and the objects’ similarities in the proposed SE-Plane for different objects are presented in Section 3; and the experiments, discussion, and conclusions are given in Section 4 and Section 5, respectively.

## 2. Related Work

### 2.1. Objects Similarity

#### 2.1.1. Categories and Shapes

**Figure 1:**
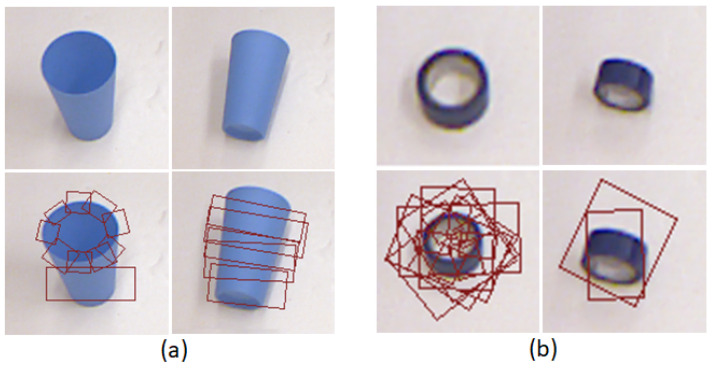
Two objects selected from Cornell dataset, which have different shapes and grasp positions because of different poses and views.

The object shapes vary in image presentations with different poses and observation views. For example, the images in Figure 1 are from the Cornell grasping datasets [21]. In Figure 1, the images in the top and the bottom row present the objects’ shapes and the grasp position labels, respectively. The two images in the bottom row of Figure 1a show that the cup has different grasping positions between its “standing upright” (left) and “laying down” (right) states. A similar situation happened with the gummed paper tape shown in Figure 1b. This indicates that object grasp positions are largely influenced by the objects’ image presentations, which vary for their different poses and observation views. There are a few robotic grasping datasets, including Cornell [22], Jacquard [23], YCB [13], ARC [24], VMRD [25], etc. The objectss information, such as their categories, image presentations, and grasp anchors, were labeled in these datasets, but their shapes were not indicated.

#### 2.1.2. Shape Similarity

Shape similarity has been widely discussed in object retrieval [6,26], image matching [3,27,28,29,30], and robotic grasping tasks [19,21,31]. In these works, the Hu moment invariants method, proposed in 1962, was the earliest and a very classical shape descriptor [32]. Now, Hu moment is still widely concerned in objects’ shape analyzes because of its rotation and scale invariance [29,33,34].

Shape Context (SC) [3] is also a typical shape feature descriptor that is widely recognized in object matching. Since it is invariant to rotation, translation, and scaling transformation, it is also widely adopted in robot grasp tasks.

A histogram of oriented gradient (HOG), originally proposed for human detection, is an edge- and gradient-based descriptor [35]. The HOG method is suitable for the robotic grasp task, especially when the image foreground has boundaries distinguished from the background [15].

Besides Hu moments, Shape Context, and HOG methods, there are also other well-known shape similarity calculation methods, such as the Iterative Closest Point (ICP) [36], Point Distribution Model (PMP), etc. In this work, we use Hu moment, Shape Context, and HOG in the object shape similarity calculation, since they are widely used in grasping tasks.

#### 2.1.3. DNN Objects Similarity

Deep neural networks (DNNs) have been proved to be excellent tools to measure objects similarity [28]. An autoencoder is a widely recognized method for extracting effective image features for objects similarity, whereby a decoder recovers the image presentation from the low-dimensional presentation. In this way, the low-dimensional feature map could be applied for shape matching and image retrieving [17,28,37,38,39].

With the excellent abilities of data reduction and feature extraction of the autoencoder, we used an autoencoder for objects similarity estimation in this work.

### 2.2. Grasping Position Similarity

Before 2000, the policies of grasping novel objects were generally constructed according to the manipulator joint torque, the friction between the touch point and the gravity of the object [40,41,42]. In this period, grasp performance was directly evaluated on the number of trials before a successful grasp [4]. From 2000 to 2013, techniques of grasping novel objects aimed to achieve faster learning of grasp knowledge [2,7,19], more accurate detections of grasp positions [2], and a more robust grasp of an unknown object [7,43,44,45].

Since 2013, when the deep neural network (DNN) was first used in grasping tasks [9], various networks have been proposed to learn the grasp knowledge from image presentations. Inspired by object detection algorithms, such as Yolo v1–Yolo v5 [10,46,47], single-shot multi-box detection (SSD) [48] and the anchors, which were used to indicate the ranges of objects (such as the width, height, and the center point of the targets) have also been adopted to represent the grasp positions of the objects [10,49]. Despite the high performance of deep architectures on grasp position prediction, the hypothesis that similar objects have similar grasping positions has not been validated.

With the development of DNN grasp techniques, some researchers constructed grasping datasets for DNN training, such as Cornell [22], Jacquard [23], YCB [13], ARC [24], and VMRD [25]. In these datasets, the grasping positions are labeled with anchors. Intersection-over-union (IoU) between the predicted anchors and the ground truth is used as the network loss function in the training process and grasp performance evaluation. In this work, we use the anchors provided by the datasets and IoU to evaluate the grasping similarity between different objects.

### 2.3. Objects’ Similarity and Grasping Positions Similarity

Just as mentioned in Section 2.1, the classical methods such as Hu moment, Shape Context, HOG, etc., are able to describe objects’ shape features very well. In addition to these, the autoencoder is able to reduce the dimensions of objects’ shape features for similarity evaluation. We have also introduced that IoU between the anchors of two objects implies their grasp similarities. However, the relevance between the similarity of the objects and grasp similarity has not been discussed before. This is just the issue our work will focus on.

## 3. Definitions

We aim to find the relationship between objects similarity and grasp similarity. In this section, we first introduce the related conceptions and definitions. **Similarity-estimation Plane (SE-Plane):** Let $obj_{i}\left(1\leq i\leq m\right)$ be the ith object in a grasp evaluation dataset *X*, where *m* is the total number of objects in *X*. $os\left(i,j\right)$ and $gs\left(i,j\right)$ are the normalized values of objects similarity and the grasp similarity between $obj_{i}$ and $obj_{j}$, where $0.0\leq os\left(i,j\right)$, $gs\left(i,j\right)\leq 1.0$ and larger $os\left(i,j\right)$ and $gs\left(i,j\right)$ imply higher objects similarity and grasp similarity between $obj_{i}$ and $obj_{j}$.

Supposing that $\Gamma_{i}^{os}=\left\{os\left(i,1\right),\ldots ,os\left(i,j\right),\ldots ,os\left(i,m\right)\right\}$ is the set of objects similarity, where all the items are sorted in reverse order with os(i,1)≥⋯≥os(i,j)≥⋯≥os(i,m), the number $j_{k}^{os}$ is the index of the object which is the $k^{\mathrm{th}}$ maximal item in $\Gamma_{i}^{os}$.

Given a two-dimensional (2D) coordinate plane ${XY}_{Os-Gs}$, where the *x* coordinate records the values of $os(i,j_{k}^{os})$, and those of the *y* coordinate present the corresponding values of $gs(i,j_{k}^{os})$. In this way, the point $p(os\left(i,j_{k}^{os}\right),gs\left(i,j_{k}^{os}\right))$ presents a 2D point in the ${XY}_{Os-Gs}$ plane, whose *x* value presents the top $k^{\mathrm{th}}$ maximal os(i,j) values for ${obj}_{i}$, and the *y* value presents the corresponding values of gs(i,j) for ${obj}_{i}$ and ${obj}_{j}$, where $j=j_{k}^{os}$. In this work, we also call the 2D plane ${XY}_{Os-Gs}$ the similarity-estimation plane (SE-Plane).

**Figure 2:**
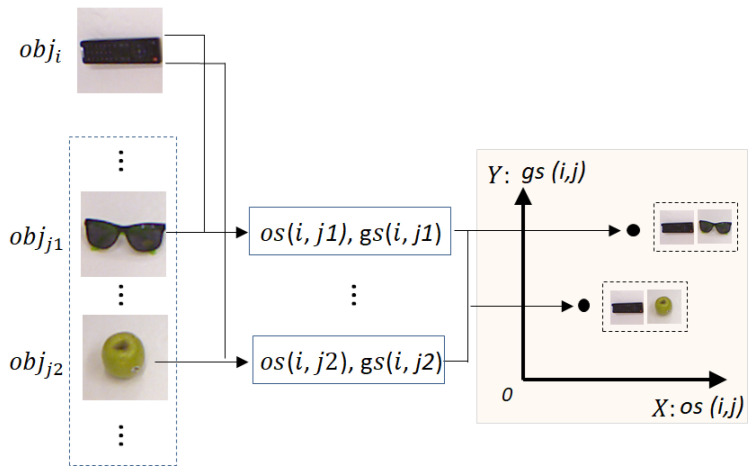
An example of the relationship between grasp similarity and objects similarity of two different object pairs.

Figure 2 shows a sample, which presents the relationship of the objects similarity (os) and grasp similarity (gs) in the SE-Plane for two object pairs. In Figure 2, $obj_{i}$, $obj_{j1}$, and $obj_{j2}$ are a remote control, sunglasses, and an apple, respectively. Intuitively, $obj_{i}$ and $obj_{j1}$ have higher similarity (rectangle vs. dumbbell shape) than $obj_{i}$ and $obj_{j2}$ (rectangle vs. circle). Then, it is expected that $p(os\left(i,j_{1}\right),gs\left(i,j_{1}\right))$ is located at the upper right side of $p(os\left(i,j_{2}\right),gs\left(i,j_{2}\right))$ in the SE-Plane, just as presented in Figure 2. Figure 2 gives a simple and straightforward sample for two assumed object pairs in the proposed 2D $XY_{Os-Gs}$ SE-Plane. Based on the definition of the SE-Plane, we have two inferences:

**Inference** **A:** If the assumption “do similar objects have similar grasp positions” was true, then the 2D point of two objects in the SE-Plane, which has higher similarity for their object and grasp position, should be located at the top-right area of the points whose similarities are smaller.

**Inference** **B:** It is also expected that given some common objects $obj_{i}\left(1\leq i\leq m\right)$, which are randomly picked from the open, widely recognized grasping datasets, the points $p\left(os\left(i,j_{k}^{os}\right),gs\left(i,j_{k}^{os}\right)\right)$ with smaller *k* should be located at the top-right area of those with larger *k*. In the experiment section, we will analyze and discuss whether Inferences A and B could be proved using the classical objects’ similarity evaluation methods (including the shapes similarity strategies) and the grasping similarity obtained by the IoU between the grasping anchors in the open datasets. Furthermore, if Inferences A and B were correctly proved, it is reasonable that the answer to the question “do similar objects have similar grasp positions?” is true. In addition, we hope to find some valuable patterns between grasping positions and their objects’ similarities as well.

## 4. Experiments

Before the experiments, we first outlined the methods for objects similarity, grasp positions similarity evaluation, the grasping datasets, and the objects used in experiments as well.

### 4.1. Objects Similarity

#### 4.1.1. Hu Moment Invariants

For a given $obj_{i}$, we adopted the seven values of Hu moment as the objects similarity vector, which is denoted as $Hu_{i}\left(s\right)\left(1\leq s\leq S\right)$. We used the function HuMoments() provided by the open computer vision library (OpenCV) [50] to obtain the seven Hu moments from an object image. Then, the Hu objects similarity between $obj_{i}$ and $obj_{j}$ was obtained by (1), where ${\hat{Hu}}_{i,j}\left(s\right)$ was used to normalize the $s^{th}$ Hu moment pair to the range of $\left(0,1\right)$ for $obj_{i}$ and $obj_{j}$, and $os_{Hu}\left(i,j\right)$∈ (0.0, 1.0). The larger value of $os_{Hu}\left(i,j\right)$ implies the higher objects similarity between $obj_{i}$ and $obj_{j}$ on the Hu moment measurement.

(1) $$os_{Hu}\left(i,j\right)=\sum_{s=1}^{S}\left(1-\left.\left|\right|{\hat{Hu}}_{i,j}\left(s\right)\right.\left|\right|\right){,where\;\hat{Hu}}_{i,j}\left(s\right)=\frac{||Hu_{i}\left(s\right)\left|\right|-||Hu_{j}\left(s\right)\left|\right|}{max\left(Hu_{i}\left(s\right),Hu_{j}\left(s\right)\right)}$$

#### 4.1.2. Shape Context

Shape Context (SC) uses log-polar histogram bins to describe contour features. $h_{i}\left(k\right)$ denotes the object feature descriptor from one object *i* [3]. The minimum $H\left(\pi \right)$ implies the correlation coefficient of $obj_{i}$ and $obj_{j}$ according to Equation (2). The matching cost of two objects *i*, *j* under a possible feature matching π is denoted as Equation (3). In Equation (3), $C_{i,j}$ reflect the cost between contour points $p_{i}$ and $q_{j}$.

(2) $$os_{SC}\left(i,j\right)=min\left(H\left(\pi \right)\right)$$

(3) $$H\left(\pi \right)=\sum_{i}C\left(p_{i},q_{\pi \left(i\right)}\right){,where\;C}_{i,j}=C\left(p_{i},q_{j}\right)=\frac{1}{2}\sum_{k=1}^{K}\frac{{\left[h_{i}\left(k\right)-h_{j}\left(k\right)\right]}^{2}}{h_{i}\left(k\right)+h_{j}\left(k\right)}$$

#### 4.1.3. Histogram of Oriented Gradient (HOG)

We used the function HOGDescriptor() provided by OpenCV to obtain the HOG vector $HOG_{i}\left(s\right)$ (1≤s≤S) for $obj_{i}$, where *S* is the length of the HOG vector. The correlation coefficient of the HOG similarity between $obj_{i}$ and $obj_{j}$ is presented as Equation (4). Compared with Hu moments, whose features contain only seven items, the length of the HOG feature vector is far larger than that of the Hu moments. In this case, the correlation coefficient is more suitable to describe whether two curves have consistency in their change tendency. The correlation coefficient of the HOG similarity between $obj_{i}$ and $obj_{j}$ is presented in Equation (4).

(4) $$os_{HOG}\left(i,j\right)=\frac{\sum_{s}\left(\left(HOG_{i}\left(s\right)-\bar{HOG_{i}}\right)\left(HOG_{j}\left(s\right)-\bar{HOG_{j}}\right)\right)}{\sqrt{\sum_{s}{\left(HOG_{i}\left(s\right)-\bar{HOG_{i}}\right)}^{2}}\sqrt{\sum_{s}{\left(HOG_{j}\left(s\right)-\bar{HOG_{j}}\right)}^{2}}}$$

#### 4.1.4. Autoencoder

We also used a deep full-connection (FC) neural network [51] and CNN structure autoencoders to convert object images to a low-dimensional feature map for objects similarity evaluation. Different from the Hu, Shape Context, and HOG features, features obtained by the FC network and CNN autoencoder tend to be implicit. We used the Euler distances to calculate the similarity between the feature vectors obtained by them.

(5) $$\;os_{FC}\left(i,j\right)=1-\frac{\sqrt{\sum_{s}{\left(Auto_{i}^{FC}\left(s\right)-Auto_{j}^{FC}\left(s\right)\right)}^{2}}}{\sqrt{\sum_{s}{\left(Auto_{i}^{FC}\left(s\right)\right)}^{2}}\sqrt{\sum_{s}{\left(Auto_{j}^{FC}\left(s\right)\right)}^{2}}}$$

(6) $$os_{CNN}\left(i,j\right)=1-\frac{\sqrt{\sum_{s}{\left(Auto_{i}^{CNN}\left(s\right)-Auto_{j}^{CNN}\left(s\right)\right)}^{2}}}{\sqrt{\sum_{s}{\left(Auto_{i}^{CNN}\left(s\right)\right)}^{2}}\sqrt{\sum_{s}{\left(Auto_{j}^{CNN}\left(s\right)\right)}^{2}}}$$

We denoted the low-dimensional feature maps obtained by the FC network and CNN autoencoder for $obj_{i}$ with $Auto_{i}^{FC}\left(s\right)$ and $o_{i}^{CNN}\left(s\right)$$\left(1\leq s\leq S\right)$, then we used (5) and (6) to calculate the objects similarity between $obj_{i}$ and $obj_{j}$.

### 4.2. Grasp Similarity

The grasp similarity between two objects can be quantified by calculating the IoU between their labeled anchors. Considering that each object has multiple grasping anchors, we adopted two methods to obtain the grasp similarity for $obj_{i}$ and $obj_{j}$ in this work, average grasp similarity, $gs_{A}\left(i,j\right)$, and the maximal grasp similarity, $gs_{M}\left(i,j\right)$.

(7) $$gs_{A}\left(i,j\right)=\frac{\sum_{s}{\bar{gs}}_{A}\left(i,j\right)}{S},where\;{\bar{gs}}_{A}\left(i,j\right)=\frac{a_{s}^{IoU}\cap b_{s}^{IoU}}{a_{s}^{IoU}\cup b_{s}^{IoU}}$$

(8) $$gs_{M}\left(i,j\right)=\vee_{s}max\left(\frac{a_{s}^{IoU}\cap b_{s}^{IoU}}{a_{s}^{IoU}\cup b_{s}^{IoU}}\right)$$

Let $a_{i}\left(1\leq i\leq I\right)$ and $b_{j}\left(1\leq j\leq J\right)$ be the anchors of $obj_{i}$ and $obj_{j}$, and *I* and *J* are the anchor numbers of $obj_{i}$ and $obj_{j}$, respectively. $a_{s}^{IoU}$ and $b_{s}^{IoU}\;\left(1\leq s\leq S\right)$ are the interacted anchors of $obj_{i}$ and $obj_{j}$ when both their centers and main axes coincide. $S\leq min\left(I,J\right)$ is the number of interacted anchors for $obj_{i}$ and $obj_{j}$. Then $gs_{A}\left(i,j\right)$ and $gs_{M}\left(i,j\right)$ can be obtained by (7) and (8), respectively.

### 4.3. Data Preparation

In this work, we carried out experiments both on public grasping datasets (Cornell [22] and Jacquard [23]) and the image presentations from the realistic objects.

**Figure 3:**
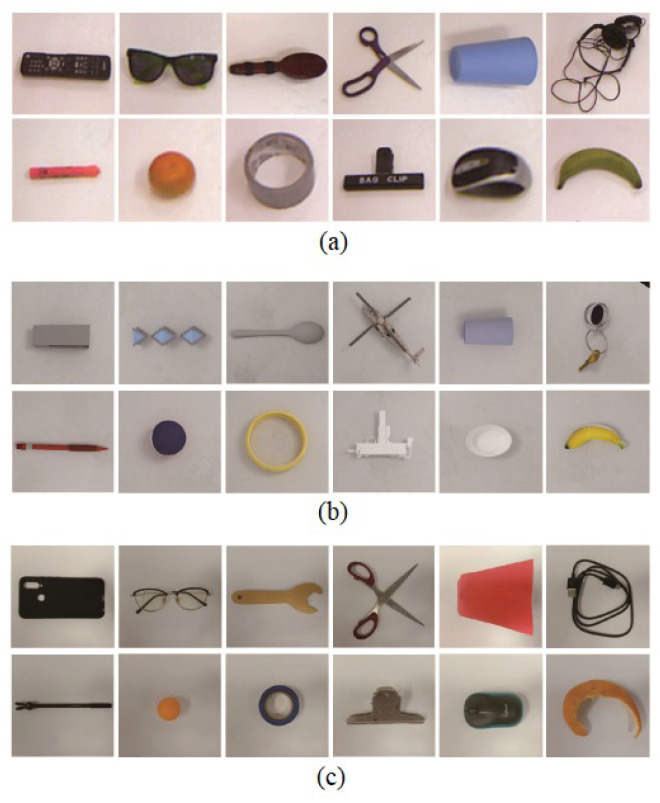
The image presentations for the objects with commonly seen shapes in experiments. (**a**) 12 objects from Cornell dataset; (**b**) 12 objects from Jacquard dataset; (**c**) 12 realistic objects from the dataset constructed in this work.

The Cornell grasping dataset contains 885 images of 240 grasp objects, and Jacquard contains 54 k images of 11 k objects. The labels are presented with grasp anchors. Inspired by the pioneering work proposed in [18], we selected the objects with shape primitives to analyze the relationship between grasp similarity and objects similarity in experiments. In [18], the authors divided the grasping shape primitives into 5 categories: Boxes, Spheres, Cylinders (Side Grasp), Cylinders (End Grasp), and Cones. In this work, we first selected 12 objects from the Cornell dataset which are listed in Figure 3a. These 12 objects cover the common shapes of the rectangle, dumbbell, fusiform, cross-shaped, trapezoid, strip, circle, ring-shaped, T-shape, ellipse, arc, and irregular shapes. The number of primitive shapes discussed in this work is 12, which is far more than the 5 primitive shapes discussed in [18]. Similarly, we also selected 12 objects with the same primitive shapes from the Jacquard dataset (Figure 3b). These 24 objects were used to evaluate Inferences A and B.

We also constructed a dataset which contains 2080 images for 21 realistic objects. These realistic objects have the similar primitive shapes to those objects selected from Cornell [22] and Jacquard [23], and they were taken in various angles and scales. Figure 3c presents a group of them in horizontal level which are similar to those in Figure 3a,b. We named this dataset CasiaZhuasape, and a copy of this dataset is available at the address [52].

In the Hu, Shape Context, HOG, FC network, and CNN autoencoder methods, only Hu moment and Shape Context had the rotation and scale invariance. It is necessary to align the objects on scale and rotations for the HOG, FC network, and CNN autoencoder. We extracted the objects from the original images and rotated and aligned their main axes to horizontal level. Each of them was finally put in a square picture. All the square pictures were resized to 128 × 128 pixel resolutions. The 5th and the 9th images in Figure 3a present the rotation and scale normalized images of the cup and the gummed paper tape presented in Figure 1. Meanwhile, the object grasp anchors were rotated, aligned, and resized to the appropriate positions and angles as well.

### 4.4. Experiments on Public Datasets

#### 4.4.1. Objects Similarity Evaluation

In the HOG objects similarity calculation, we used a 96 × 128 window, 16 × 16 block, 16 × 16 stride, and 8 × 8 cell to obtain the feature maps; in this way, the length of $HOG_{i}\left(s\right)$ was 57,024. In the FC network, as described in [51], we used the full connection network with a 4096-2048-1024 structure to obtain the low-dimensional features for the 128 × 128 image presentations. Then, the length of ${Auto}_{i}^{FC}\left(s\right)$ was 1024. For the CNN autoencoder objects similarity calculation, we adopted the standard VGG16 proposed in [53] for data reduction. The 128 × 128 images were converted to the feature vector at the length of 2048. The VGG16 AutoEncoder structure in this work consisted of 13 convolution layers and 3 full connection layers. The standard structure of VGG16 takes Euclidean Distance (Mean Squared Error, MSE) as loss calculation for full connection layers. Euclidean Distance is also one of the widely recognized measurement functions for the image similarity calculation method. Therefore, we used Euclidean Distance (Mean Squared Error, MSE)-based logistic regression as a loss function for VGG16 in this work.

**Figure 4:**
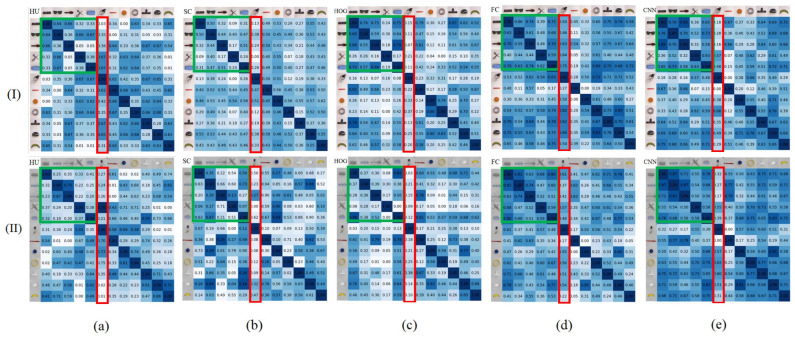
The shape similarity confusion matrixes of (**a**) $os_{Hu}\left(i,j\right)$, (**b**) $os_{SC}\left(i,j\right)$, (**c**) $os_{HOG}\left(i,j\right)$, (**d**) $os_{FC}\left(i,j\right)$, and (**e**) $os_{CNN}\left(i,j\right)$ for the objects in Figure 3, where the images in column (I) and column (II) are the objects from Cornell (Figure 3a) and Jacquard (Figure 3b), respectively.

The images in the rows of Figure 4a–e list the symmetric confusion matrixes of $os_{Hu}\left(i,j\right)$, $os_{SC}\left(i,j\right)$, $os_{HOG}\left(i,j\right)$, $os_{FC}\left(i,j\right)$, and $os_{CNN}\left(i,j\right)$, where the images in the columns of Figure 4(I) and Figure 4(II) are the results for the objects selected from Cornell and Jacquard, respectively. In Figure 4, the darker grid implies greater similarity between $obj_{i}$ and $obj_{j}$. We can see from Figure 4 that these symmetric confusion matrixes are similar in the distribution of the values, especially the values in the green rectangle areas. Furthermore, the values of OS among the first three objects seem relatively higher. In these symmetric matrixes, the general colors of $os_{Hu}\left(i,j\right)$, $os_{SC}\left(i,j\right)$, and $os_{HOG}\left(i,j\right)$ are relatively lighter than those of $os_{FC}\left(i,j\right)$ and $os_{CNN}\left(i,j\right)$. One possible reason is that the SC, Hu, and HOG pay more attention to the gradient features, while the FC network and CNN autoencoder obtained the objects’ shape and appearance features synchronously. In this way, the symmetric matrixes can be divided into two groups: Group A (SC, Hu, and HOG) for shape, and Group B (FC network and CNN autoencoder) for appearance.

In Group A, the HOG method outperformed the SC and Hu methods in the objects similarity evaluation. For example, the first object (a remote control in column I and a rectangle box in column II) and the 5th object (two cups in the shape of a trapezoid) in Cornell and Jacquard had high shape similarity to the perspective of humanity, and results of the HOG showed exactly that object 1 and 5 have higher OS values, while the corresponding OS values of the SC and Hu were relatively smaller. In Group B, the FC and CNN methods obtained similar OS distribution, which illustrates that the deep learning method can generally extract more effective features. Moreover, the CNN method tends to have better performance on similarity distinguishing. For example, in the values in the red rectangle areas, the 6th objects (the two earphone wires in irregular shapes) were very different from other objects in the perspective of humanity, and the results of the CNN had lower OS values than the FC between the 6th objects and the others. Therefore, in the following experiments, the HOG and CNN were applied for further analysis since they are the better ones in Groups A and B.

#### 4.4.2. Objects Similarity and Grasping Positions Similarity

**Figure 5:**
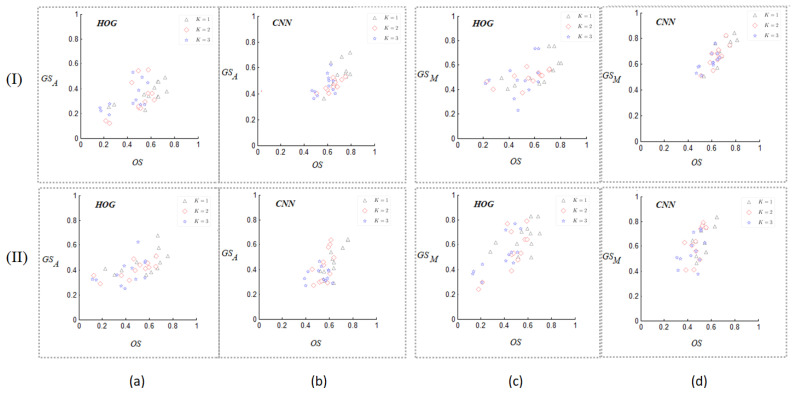
All the top *k* points for the HOG and CNN autoencoder with 1≤k≤3 in the SE-Plane, respectively, for the objects listed in Figure 3a,b: (I) the objects from the Cornell dataset (Figure 3a); (II) the objects from the Jacquard dataset (Figure 3b).

Figure 5a,b list all the top *k*$\left(1\leq k\leq 3\right)$ points $p\left(os\left(i,j_{k-i}^{os}\right),gs_{A}\left(i,j_{k-i}^{os}\right)\right)$ for the HOG and CNN autoencoder in the proposed SE-Plane, respectively, for the objects in Figure 3a,b. In Figure 5, the values of *X*-coordinate present the top 1, top 2, and top 3 objects similarity for $obj_{i}\left(1\leq i\leq 12\right)$, and those of *Y*-coordinate present the average grasping similarity between the selected top $j_{k}^{os}$ objects (1≤k≤3) for $obj_{i}$. Figure 5c,d list all the top k(1≤k≤3) points $p\left(os\left(i,j_{k}^{os}\right),gs_{M}\left(i,j_{k}^{os}\right)\right)$ for the HOG and CNN autoencoder.

We can see from Figure 5 that the top 1 points are mostly located in the upper right area of the top 2 points, and the top 1 and top 2 points are mostly located at the upper right area of the top 3 points. This indicates that if two objects ($obj_{i}$ and $obj_{j}$) have high objects similarity, they tend to have a high grasp similarity.

In addition, all the top 3 points $p\left(os\left(i,j_{k}^{os}\right),gs_{M}\left(i,j_{k}^{os}\right)\right)$ in Figure 5c,d are more distinguishing compared with the corresponding points in Figure 5a,b. This indicates that $gs_{M}\left(i,j\right)$ have better distinction than $gs_{A}\left(i,j\right)$. At the same time, the points in Figure 5b,d have better linearity than those in Figure 5a,c. This indicates that the objects similarity obtained by the CNN autoencoder has better linear correlation with the grasp similarity than that obtained by HOG.

In general, the object pairs with higher objects similarity and grasping similarity are located at the upper right area of the objects with lower objects and grasping similarity. Namely, the points with smaller *k* are located at the top-right areas of the ones with larger *k*. This shows that the Inference *B* is reasonable and correct.

#### 4.4.3. The Patterns for Objects Similarity and Grasping Positions

**Figure 6:**
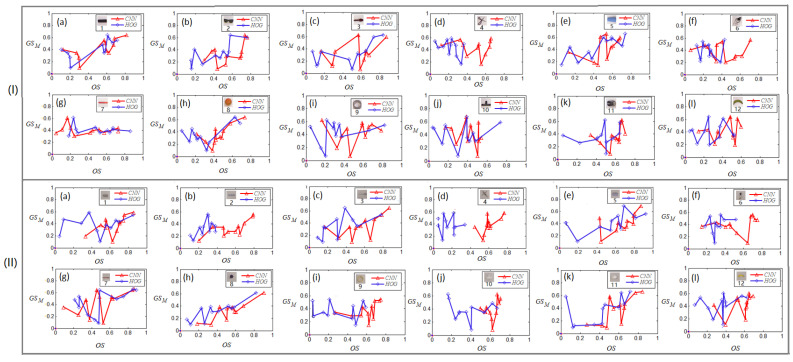
The curves of the relationship between the max IoU values of the grasping positions and the objects similarity for each object in Figure 3a,b. (I) The objects from the Cornell dataset (Figure 3a); (II) the objects from the Jacquard dataset (Figure 3b).

The 24 images in Figure 6 (I) and (II) list the values of $\Gamma_{i}^{os}$ in the SE-Plane between the max IoU values of the grasping positions and the objects similarity for each object given in Figure 3a,b, where the connecting line in red means that the values of objects similarity were obtained by the CNN autoencoder, and the ones in blue were from the HOG. We can see from Figure 6 that the points which have the highest values of OS are mostly located at the top-right areas of the SE-Plane, and the points with higher similarities in object and grasp positions are mostly located in the top-right area of the points whose object and grasp position similarities are relatively smaller, especially the points labeled by red ‘Δ’ and blue “◊” in Figure 6(I)(II)(a–c,e,h–l).

In addition, we can infer from Figure 6(I)(II)(a–c,e,g,h,k) that the red curves in these small charts have clear upward trends from the bottom-left to the top-right. This indicates the grasping positions of these objects, namely the rectangle (Figure 6(I)(II)(a)), dumbbell (Figure 6(I)(II)(b)), fusiform (Figure 6(I)(II)(c)), trapezoid (Figure 6(I)(II)(e)), trip (Figure 6(I)(II)(g)), sphere (Figure 6(I)(II)(h)), and ellipse (Figure 6(I)(II)(k)), are obviously positive correlations to the objects’ similarities. In this way, we can infer that given an unknown object, if it is similar to the ones listed in Figure 6(I)(II)(a–c,e,g,h,k) in shape or appearance, then its grasping positions are reasonably similar to them. On the other hand, for the curves in Figure 6(I)(II)(d,f,i–l), regardless of the red or blue curves, the value changes of GS had less relationships with those of the OS. This indicates that the shapes, namely the cross (Figure 6(I)(II)(d)), irregular (headphone cable in Figure 6(I)(II)(f)), loop (Figure 6(I)(II)(i)), T-type (Figure 6(I)(II)(j)), and arc (Figure 6(I)(II)(l)), are not suitable as grasp references for unknown objects. In general, the shapes listed in Figure 6(I)(II)(a–c,e,g,h,k), namely the shapes of the rectangle, dumbbell, fusiform, trapezoid, strip, sphere, and ellipse, have better performances to measure the unknown objects’ grasping positions through evaluating their similarities to the unknown object in the proposed SE-Plane.

### 4.5. Experiments on Realistic Objects

#### 4.5.1. Objects Similarity

**Figure 7:**
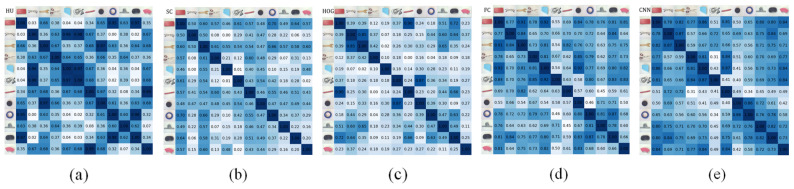
The objects similarity confusion matrixes of (**a**) $os_{Hu}\left(i,j\right)$, (**b**) $os_{SC}\left(i,j\right)$, (**c**) $os_{HOG}\left(i,j\right)$, (**d**) $os_{FC}\left(i,j\right)$, and (**e**) $os_{CNN}\left(i,j\right)$ for the realistic objects listed in Figure 3c.

Figure 7 lists the objects similarity confusion matrixes of $os_{Hu}\left(i,j\right)$, $os_{SC}\left(i,j\right)$, $os_{HOG}\left(i,j\right)$, $os_{FC}\left(i,j\right)$, and $os_{CNN}\left(i,j\right)$ for the 12 selected realistic objects listed in Figure 3c. The general colors of $os_{Hu}\left(i,j\right)$, $os_{SC}\left(i,j\right)$, and $os_{HOG}\left(i,j\right)$ are relatively lighter than those of $os_{FC}\left(i,j\right)$ and $os_{CNN}\left(i,j\right)$. We also divided these symmetric matrixes into Group A (SC, Hu and HOG) and Group B (FC network and CNN autoencoder).

Just like the results presented in Figure 4, in Group A, the HOG represented better performance on objects similarity, which coincided more with the perspective of humanity (e.g., the OS relevance among the first, second, and the third object, and the OS differences for the 4th and the 6th object with others). Similarly, the FC and CNN methods in Group B also obtained similar reliable OS distribution to those in Figure 4, while the CNN method performed better.

These experiments on the images of realistic objects reveal that these evaluation methods are still reliable in physical environments.

#### 4.5.2. Grasp Similarity

**Figure 8:**
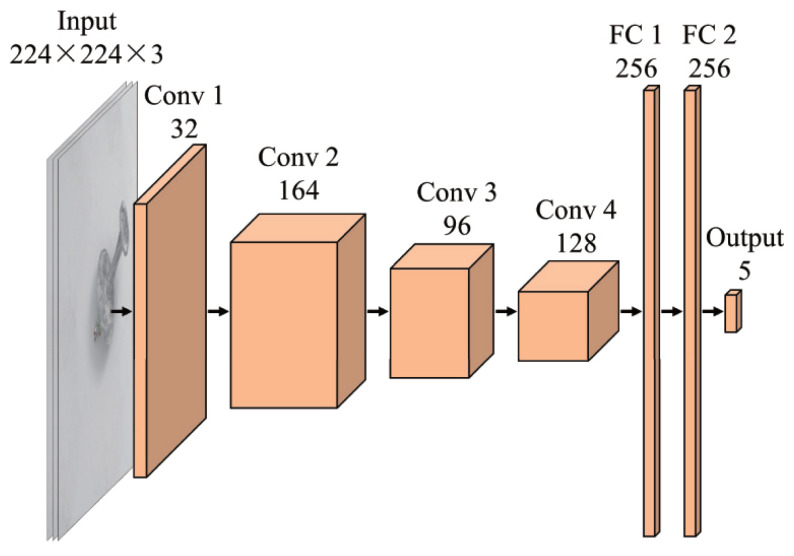
The backbone structure of the network used for grasp detection.

**Figure 9:**
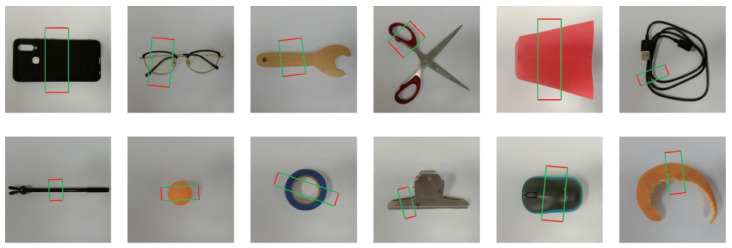
The predicted grasp anchors of the objects given in Figure 3c.

Since the objects in the realistic dataset have no pre-labeled grasp anchors, we adopted the grasp prediction network (GP-Net) described in [54] to generate multiple possible grasp anchors for these objects. This method achieved an accuracy of 94.8% with a speed of 74.07 fps on the Cornell grasping dataset on GeForce GTX 1050 Ti; therefore, it is reliable enough to predict grasp anchors in this work.The network received an RGB image the size of 224 × 224 × 3 as input and generated encoded grasp *x*, *y*, *w*, *h*, θ, where *x*, *y*, *w*, *h*, θ are the center point, width, height, and rotation of the predicted grasp anchor. The 128 × 128 resolution images were resized to 224 × 224 before they were inputted into the GP-Net. Figure 8 presents its structure, which contained 4 convolutional layers and 2 FC layers. The network was trained on the mixed datasets of Cornell and Jacquard, and a pre-trained model of VGG16 on MS COCO [55] was applied to accelerate the training. Figure 9 lists the predicted grasp anchors for each object given in Figure 3c. For more prediction results, please refer to the address at [52]. These grasp anchors were used to calculate the maximal grasp similarity $gs_{M}\left(i,j\right)$ as Equation (8).

#### 4.5.3. Objects Similarity and Grasping Positions Similarity

**Figure 10:**
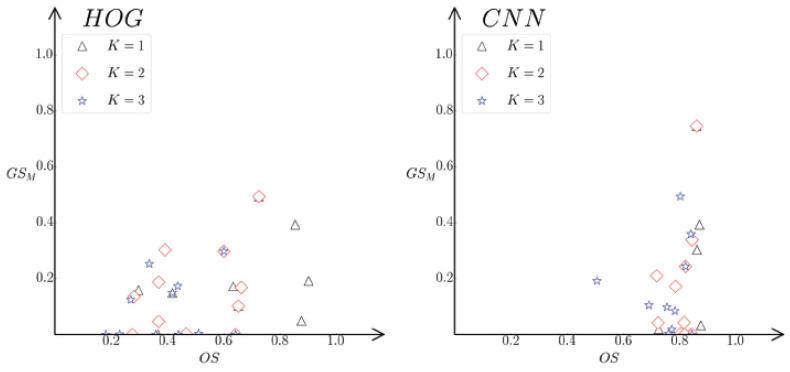
The top k(1≤k≤3) points $p(os\left(i,j_{k}^{os}\right),gs_{M}\left(i,j_{k}^{os}\right))$ for the HOG and CNN autoencoder in the SE-Plane, respectively, for the objects listed in Figure 3c.

Similar to Figure 6, Figure 10 lists the top k(1≤k≤3) points $p\left(os\left(i,j_{k}^{os}\right),gs_{M}\left(i,j_{k}^{os}\right)\right)$ for the HOG and CNN autoencoder in the SE-Plane, respectively, for the objects in the realistic objects. It is noticeable that the values of grasp similarity for the realistic objects are generally smaller than those in the public dataset. This is because only the top five grasp anchors were used for each realistic object, while at least ten or more grasp anchors were always labeled for each object in the public dataset.

We can see from Figure 10 that the data distributions of the realistic objects are similar to those in the public dataset. The top 1, top 2, and top 3 points are generally distributed from the upper-right to the lower-left of the SE-Plane, which indicates that objects with high objects similarity tend to have a high grasp similarity.

In addition, similar to the results in the public dataset given in Figure 5, the results for realistic objects also show that the CNN method has better performance on objects similarity evaluation because the data distribution of the CNN has better linearity along the bottom-left to top-right than those of the HOG.

## 5. Discussions

In experiments, we firstly compared the objects similarity performances obtained by the Shape Context, Hu moment, HOG, FC network, and CNN autoencoder on the objects selected from the Cornell and Jacquard grasping datasets. It was found that the HOG and CNN autoencoder have better distinguishing performance than those of the SC, Hu, and FC network.

Furthermore, we adopted the HOG and CNN autoencoder in the similarity analysis between the objects similarities and the similarities of their grasping positions by converting the relative objects pairs into the SE-Plane. It was found that the 2D point of two objects with both higher objects similarity and grasp similarity are located at the upper right area of the points with lower objects similarities and grasps similarities. In addition, the points with smaller k(1≤k≤3) are located at the upper right area of those with larger *k*. It proves that objects with higher objects similarity generally have higher grasp similarity. These situations proved that the more similar two objects, the more similar the grasping positions for them. In this way, Inference A and Inference B were confirmed, and the answer to the question “Do Similar Objects Have Similar Grasp Positions?” is that this is reasonably correct. We also found that the points obtained by maximal IoU grasping similarity were better clustered in the SE-Plane than those of the average IoU grasping similarity. It reveals that the maximal IoU has better performance on analyzing the relationship between grasping similarity and objects similarity than the average IoU.

Finally, we used the maximal IoU to evaluate the relationship between the objects similarity and grasping similarity of a single object. We found that grasping similarity is generally positively correlated to objects similarity. This is also intuitive evidence to prove that “Do Similar Objects Have Similar Grasp Positions?” is true. Furthermore, it was found that the CNN autoencoder had higher performance in the objects similarity evaluation for the correlation analysis between the grasping similarity and objects similarity than the HOG. In addition, it was also found that the objects with better positive correlation between the CNN’s objects similarity and grasp similarity were more suitable to be regarded as primitive shape objects, such as the rectangle, dumbbell, fusiform, trapezoid, strip, sphere, loop, and ellipse.

## 6. Conclusions

In this work, we aimed to confirm that “similar objects have similar grasp positions” is correct by analyzing the relationship between the object shape similarity and grasp positions similarity. To this end, we proposed the SE-Plane, whose horizontal and vertical axes indicate the objects similarity and grasp similarity, respectively. Then, the proof of the issue was converted to confirm the point relationships in the SE-Plane, namely the points with higher objects similarity accordingly having higher grasp similarity. Several widely recognized shape calculation algorithms and deep learning methods ertr applied to evaluate objects similarity, and the IoU of grasp anchors was applied to evaluate grasping similarity. With experiments on the well-known public grasp datasets (Cornell, Jacquard) and realistic datasets, the inferences raised in this work were proved to be correct from the statistical results: positive correlation exists between the grasp similarity and objects similarity. We also obtained valuable information from the experiments: (1) the HOG and CNN autoencoder are relatively more valuable in objects similarity evaluation; (2) the maximal IoU of grasping anchor has better performance in grasp similarity evaluation than the average IoU; (3) the shapes, including rectangle, dumbbell, fusiform, trapezoid, strip, sphere, loop, and ellipse, have better positive correlation between shape similarity and grasping similarity. They are reliable as primitive shapes and references to measure the grasp positions for a novel object, as long as this unknown object has a similar shape to the primitive ones in the SE-Plane; (4) finally, the proposed SE-Plane presents a new strategy to measure the relationship between objects similarity and grasp similarity, thus it provides a new strategy to predict the grasping anchors for a novel object from the known objects. As long as the novel object has a similar shape or appearance to an object with the previous primitive shapes in the proposed SE-Plane, then the grasp knowledge (namely the grasp positions) could be potentially transferred to the novel object from the objects with primitive shapes. How the proposed SE-Plane could be used in the task of robotic grasp for novel objects is out of the scope of this work; we will discuss it in the future.

## Data Availability

Dataset available on request from the authors.
